# Nested case–control study of the effects of non-steroidal anti-inflammatory drugs on breast cancer risk and stage

**DOI:** 10.1054/bjoc.2000.1119

**Published:** 2000-06-02

**Authors:** C R Sharpe, J-P Collet, M McNutt, E Belzile, J-F Boivin, J A Hanley

**Affiliations:** Centre for Clinical Epidemiology and Community Studies, Sir Mortimer B Davis–Jewish General Hospital, 3755 Chemin de la Côte Ste Catherine, Montréal, Québec, H3T 1E2, Canada; Joint Departments of Epidemiology and Biostatistics and of Occupational Health, McGill University, Montréal, Québec, Canada; Population Health Branch, Saskatchewan Health, Regina, Saskatchewan, Canada

**Keywords:** breast neoplasms, anti-inflammatory agents, non-steroidal, epidemiology, case–, control studies

## Abstract

We carried out a nested case–control study to measure the rate ratio (RR) for invasive female breast cancer in relation to non-steroidal anti-inflammatory drug (NSAID) use. The source population consisted of the female beneficiaries of the Saskatchewan Prescription Drug Plan from 1981 to 1995 with no history of cancer since 1970. Four controls/case, matched on age and sampling time, were randomly selected. Dispensing rates during successive time periods characterized NSAID exposure. RRs associated with exposure during each period were adjusted for exposure during the others. Confounding by other determinants was studied in analyses adjusted with data obtained by interviewing samples of subjects accrued from mid-1991 to mid-1995. We accrued 5882 cases and 23 517 controls. Increasing NSAID exposure 2–5 years preceding diagnosis was associated with a trend towards a decreasing RR (*P* -trend = 0.003); for the highest exposure level RR = 0.76, 95% confidence interval 0.63–0.92. This protective effect could not be attributed to confounding by other determinants. In analyses involving only the cases, NSAID exposure 2–5 and 6–10 years preceding diagnosis was associated with significantly reduced risks of presenting with a large tumour (> 5 cm diameter) or distant metastasis, but not regional lymph node metastasis. The use of NSAIDs may retard the growth of breast cancers and prevent distant metastasis. © 2000 Cancer Research Campaign


*British Journal of Cancer* (2000) **83**(1), 112–120
© 2000 Cancer Research Campaign
DOI: 10.1054/ bjoc.2000.1119, available online at http://www.idealibrary.com on IDEAL®

### Nested case–control study of the effects of non-steroidal anti-inflammatory drugs on breast cancer risk and stage

**CR Sharpe[1,2], J-P Collet[1,2], M McNutt[3], E Belzile[1], J-F Boivin[1,2] and JA Hanley[2]**

[1]Centre for Clinical Epidemiology and Community Studies, Sir Mortimer B Davis–Jewish General Hospital, 3755 Chemin de la Côte Ste Catherine, Montréal, Québec, Canada H3T 1E2; [2]Joint Departments of Epidemiology and Biostatistics and of Occupational Health, McGill University, Montréal, Québec, Canada; [3]Population Health Branch, Saskatchewan Health, Regina, Saskatchewan, Canada

**Summary** We carried out a nested case–control study to measure the rate ratio (RR) for invasive female breast cancer in relation to non-steroidal anti-inflammatory drug (NSAID) use. The source population consisted of the female beneficiaries of the Saskatchewan Prescription Drug Plan from 1981 to 1995 with no history of cancer since 1970. Four controls/case, matched on age and sampling time, were randomly selected. Dispensing rates during successive time periods characterized NSAID exposure. RRs associated with exposure during each period were adjusted for exposure during the others. Confounding by other determinants was studied in analyses adjusted with data obtained by interviewing samples of subjects accrued from mid-1991 to mid-1995. We accrued 5882 cases and 23 517 controls. Increasing NSAID exposure 2–5 years preceding diagnosis was associated with a trend towards a decreasing RR (*P*-trend = 0.003); for the highest exposure level RR = 0.76, 95% confidence interval 0.63–0.92. This protective effect could not be attributed to confounding by other determinants. In analyses involving only the cases, NSAID exposure 2–5 and 6–10 years preceding diagnosis was associated with significantly reduced risks of presenting with a large tumour (> 5 cm diameter) or distant metastasis, but not regional lymph node metastasis. The use of NSAIDs may retard the growth of breast cancers and prevent distant metastasis. © 2000 Cancer Research Campaign

**Keywords:** breast neoplasms; anti-inflammatory agents, non-steroidal; epidemiology; case–control studies

Non-steroidal anti-inflammatory drugs (NSAIDs) interfere with the growth of mammary tumours (Lala et al, 1997; Robertson et al, 1998) and prevent distant metastasis (Hubbard et al, 1988; Khoo et al, 1992) in rodents. The epidemiologic evidence for similar effects in women is limited and inconsistent (Isomäki et al, 1978; Friedman and Ury, 1980; Gridley et al, 1993; Thun et al, 1993; Schreinemachers and Everson, 1994; Harris et al, 1995, 1996; Egan et al, 1996).

To study the effects of NSAID use on breast cancer risk we carried out a nested case–control study, using the records of the Saskatchewan Cancer Agency (SCA) and data collected routinely by the Saskatchewan Prescription Drug Plan (SPDP), which has provided full or partial outpatient drug coverage to the Saskatchewan population since 1975.

To study the effects of NSAID use on breast cancer growth and spread we carried out analyses using the drug exposure histories of the cases and attributes of the stage of their tumours at diagnosis, as assessed by the international tumour–lymph node–metastasis (TNM) system (Spiessl et al, 1992).

*Received 15 November 1999*
*Revised 4 February 2000*
*Accepted 4 February 2000*

*Correspondence to:* CR Sharpe

### SUBJECTS AND METHODS

### Study populations

The source population was the open population of women aged ≥ 35 years, eligible to receive benefits from the SPDP during 1981 to mid-1995 with no history of cancer since 1970 other than non-melanoma skin cancer or cervical carcinoma in situ.

Subjects entered the source population on 1 January 1981, their 35th birthday, or on the date of immigration to Saskatchewan, whichever occurred latest. Subjects left the source population on 30 June 1995, or on the date of diagnosis of breast cancer, death or emigration, whichever occurred first.

The SPDP has provided coverage for outpatient prescription drugs for 94% of the Saskatchewan population (1.01 million people in mid-1991; Rawson et al, 1992). Excluded are aboriginals, military personnel, members of the Royal Canadian Mounted Police, and inmates of federal penitentiaries: they are covered by federal agencies. The accuracy of the identifying and recorded prescription information both exceed 99% (Risch and Howe, 1994).

The cases were subjects in the source population who were diagnosed with histologically proven invasive female breast cancer and reported to the SCA. Registration of cancer cases was nearly complete (Parkin et al, 1997). To be in our study, cases must have been eligible to benefit from the SPDP for ≥ 5 years before diagnosis. Hereafter, the date of diagnosis will be designated as the 'index date'.

The potential controls for each case were the subjects in the source population born within 1 year of the case, who were alive when the case was diagnosed. From the sets of potential controls, four cases were randomly selected. The date of diagnosis was assigned to each of the matched controls as their index date. Controls also must have been covered by the SPDP for ≥ 5 years before the index date.

### Linkage methods

Information in the databases of the SPDP and the SCA was linked electronically using personal health care identifiers (Rawson et al, 1992).

### Drug exposure data

Drug exposure data were obtained from the SPDP database for the period between the index date and 1 January 1976 or the coverage initiation date, whichever was later. Drug exposure histories ranged from 5 to 19.5 years in length.

The following data were extracted for each NSAID outpatient prescription dispensed: the date, class and identity of the drug (American Hospital Formulary System classification), number of pills dispensed, and strength (mg per pill). The daily dose and treatment duration were not available.

No data were available from 1 July 1987 to 31 December 1988, because the SPDP did not record most of the dispensing of drugs to individuals then (Rawson et al, 1992). The database also lacked information on drugs dispensed during hospitalizations and as samples from physicians.

### Data on potential confounders

Information on potential confounders other than those recorded in the SPDP database was obtained from telephone interviews of selected subsamples of cases and controls with index dates between 30 June 1991 and 30 June 1995 to maximize the number of living subjects. If they died before the interview, we attempted to interview a family member.

We employed a two-stage design (Cain and Breslow, 1988). To make this phase of the study maximally informative, the stage 1 population was restricted to subjects aged 45–79 years on the index date who were eligible to benefit from the SPDP for ≥ 10 years. Subsamples, comprising the stage 2 subjects, were selected from this population according to both disease and exposure status to gain statistical efficiency and reduce cost.

Both unexposed subjects and those with high levels of NSAID exposure were deliberately over-represented in stage 2. We planned to interview roughly equal numbers ($n \approx 150$) in each of the four disease/exposure categories: the ‘balanced design’.

The selection bias introduced by subsampling according to both disease and exposure status was corrected in the analysis with the known sampling fractions. This increased the precision of the effect estimates, since the sampling fractions contained information from stage 1 about the exposure–disease relationship (Cain and Breslow, 1988).

The interviews were carried out using a questionnaire with high levels of reliability (Sharpe, 1999). The interviewers were blinded to the subjects’ disease and exposure status.

### Ethical issues and confidentiality

Letters were mailed from the SCA starting in November 1996 to obtain physicians’ consent to contact the selected cases or the families of deceased cases: 78.6% consented. Letters were then mailed from the SCA to cases requesting their informed written consent to be interviewed. Non-responders were mailed a reminder letter and another consent form. The families of deceased cases were contacted by telephone prior to mailing to ask if they would agree to receive a letter. Overall, 67.4% of the cases/families consented.

The process of contacting controls, or their families, was carried out concurrently in an identical way, except that the letters were mailed from Saskatchewan Health without requesting a physician’s consent. Overall, 43.0% of the controls/families consented.

These response rates pertained to our attempts to obtain information about *potential confounders*. We had already obtained NSAID exposure data on all subjects from the SPDP database. Response rates for exposed and unexposed subjects were very similar.

Interviews proceeded concurrently from December 1996 to April 1998. The mean interval from index date to interview was 3.7 years for cases (1.1 standard deviation (s.d.)) and 3.8 years for controls (1.1 s.d.).

Extraction of data from the databases was carried out by employees of the SCA and Saskatchewan Health. Interviews were carried out by four interviewers employed by Saskatchewan Health. The data delivered to the investigators for analysis contained no nominal information. The study was approved by ethics committees of the Cross Agency Study Committee of Saskatchewan Health, the Internal Review Board of the SCA, and the Sir Mortimer B Davis–Jewish General Hospital.

### Statistical analysis

We analysed exposure to NSAIDs as a class on the assumption that all NSAIDs would affect the risk of breast cancer similarly.

To study the effects of exposure timing we a priori divided time preceding the index date into five successive periods: 1–6 months, 7–12 months, 2–5 years, 6–10 years and 11–15 years.

Exposure during each period was characterized as the average rate of dispensing NSAIDs. The rate was based on the proportion of the recommended maximum daily dose of each different NSAID dispensed ($p_i$ = average mg day$^{-1}$ dispensed ÷ maximum mg/day recommended for NSAID$_i$) during each period. The sum of the proportions, i.e. $\Sigma p_i$ for all the NSAIDs dispensed during a period, represented the measure of exposure. The usual maximum daily dose for each drug was taken from the manufacturers’ recommendations (Krogh, 1995). The drugs and their recommended maximum daily doses are tabulated elsewhere (Collet et al, 1999, Table 1).

We did not quantify the duration of NSAID exposure because we lacked information on the length of prescribed treatment. Since NSAID use is often intermittent, we doubted the validity of estimating duration of use.

If the drug exposure history for a period was missing or incomplete, due either to the date of the subject’s entry into the source population or to the 1.5-year period of missing information, the subject was assigned to a separate category designated ‘other’ (Breslow and Day, 1980).

© 2000 Cancer Research Campaign

**Table 1** RRs of breast cancer according to NSAID exposure by time period before diagnosis, with adjustment for exposure during the other time periods. Cases and controls were accrued from 1 January 1981 through 30 June 1995

| Period before diagnosis | NSAIDs (Average daily dose[a]) | Cases *n* = 5882 | Controls *n* = 23 517 | RR[b] | 95% CI |
|---|---|---|---|---|---|
| 1–6 months | 0 | 3896 | 15961 | 1.00 | Referent |
| | $0<\Sigma p_i\leq 0.1$ | 321 | 1269 | 1.04 | 0.91–1.18 |
| | $0.1<\Sigma p_i\leq 0.3$ | 433 | 1532 | 1.11 | 0.98–1.25 |
| | $\Sigma p_i>0.3$ | 449 | 1625 | 1.05 | 0.91–1.23 |
| | *P* (trend) | | | | 0.20 |
| | Other[c] | 783 | 3130 | – | – |
| 7–12 months | 0 | 3873 | 15912 | 1.00 | Referent |
| | $0<\Sigma p_i\leq 0.1$ | 305 | 1272 | 0.99 | 0.87–1.13 |
| | $0.1<\Sigma p_i\leq 0.3$ | 435 | 1456 | 1.23 | 1.09–1.39 |
| | $\Sigma p_i>0.3$ | 457 | 1632 | 1.20 | 1.02–1.40 |
| | *P* (trend) | | | | 0.003 |
| | Other[c] | 812 | 3245 | – | – |
| 2–5 years | 0 | 1522 | 5954 | 1.00 | Referent |
| | $0<\Sigma p_i\leq 0.1$ | 1277 | 5240 | 0.93 | 0.85–1.01 |
| | $0.1<\Sigma p_i\leq 0.3$ | 311 | 1183 | 0.91 | 0.79–1.06 |
| | $\Sigma p_i>0.3$ | 197 | 848 | 0.76 | 0.63–0.92 |
| | *P*(trend) | | | | 0.003 |
| | Other[c] | 2575 | 10292 | – | – |
| 6–10 years | 0 | 1103 | 4505 | 1.00 | Referent |
| | $0<\Sigma p_i\leq 0.1$ | 1201 | 4858 | 1.01 | 0.92–1.11 |
| | $0.1<\Sigma p_i\leq 0.3$ | 235 | 912 | 1.03 | 0.87–1.21 |
| | $\Sigma p_i>0.3$ | 144 | 502 | 1.13 | 0.92–1.39 |
| | *P* (trend) | | | | 0.35 |
| | Other | 3199 | 12740 | 1.16 | 0.92–1.48 |
| 11–15 years | 0 | 938 | 3649 | 1.00 | Referent |
| | $0<\Sigma p_i\leq 0.1$ | 903 | 3793 | 0.91 | 0.82–1.01 |
| | $0.1<\Sigma p_i\leq 0.3$ | 156 | 553 | 1.03 | 0.84–1.25 |
| | $\Sigma p_i>0.3$ | 65 | 280 | 0.83 | 0.63–1.11 |
| | *P* (trend) | | | | 0.20 |
| | Other | 3820 | 15242 | 1.00 | 0.81–1.24 |

[a] See Methods. 'Other' designates the subjects with incomplete or missing exposure data for the period. [b]RRs were calculated with conditional logistic regression because of matching for age and index date. [c]RRs for this category for these periods were not estimated because of insufficient variation: since cases and controls were matched for index date, if exposure was missing for a case, due to the 1.5 year gap in the exposure histories beginning 1 July 1987, it was always missing for the matched controls, so they were all classified as 'other' and contributed no information to the matched analysis.

The 1.5 year gap in the exposure histories greatly reduced the numbers of subjects in stages 1 and 2 with known levels of exposure during the periods 2–5 and 6–10 years before the index date. Accordingly, we used the information available to impute the level of NSAID exposure. Exposure ($\Sigma p_i$) was calculated by dividing the amounts of NSAIDs known to have been dispensed during a period by the number of days on which the drugs could have been dispensed and recorded in the SPDP database. The mean numbers of days for which exposure information was available were almost identical for the two periods for the subjects in stages 1 and 2.

For analyses involving all subjects accrued from 1981 to 1995, we calculated odds ratios (ORs) with 95% confidence intervals (CIs) to estimate incidence density ratios (rate ratios (RRs)) using conditional logistic regression applied to the age- and index-date-matched case/control sets.

Because the stage 2 (interviewed) subjects were selected according to both disease and exposure status, the original matched sets were broken up. Accordingly, we calculated RRs and 95% CIs with unconditional logistic regression with adjustment for age using 5-year categories.

The statistical model used to relate the RRs to the drug exposure history was: $\exp(\beta_{1-6\text{ mos}}X_{1-6\text{ mos}}+\beta_{7-12\text{ mos}}X_{7-12\text{ mos}}+\beta_{2-5\text{ yrs}}X_{2-5\text{ yrs}}+\beta_{6-10\text{ yrs}}X_{6-10\text{ yrs}}+\beta_{11-15\text{ yrs}}X_{11-15\text{ yrs}})$ where the values of $\beta_i$ represented the regression coefficients, and the values of $X_i$ represented drug exposure during the time periods. This approach followed Miettinen's view that exposures during 'different time periods represent separate determinants … mutually confounded and thus requiring joint representation in the same model' (Miettinen, 1985).

Within each period dosage was represented by a categorical variable: unexposed (the referent), low ($0<\Sigma p_i\leq 0.1$), medium ($0.1<\Sigma p_i\leq 0.3$), high ($\Sigma p_i>0.3$), and 'other' (unknown).

The validity of the exposure imputation used for the stage 1 and stage 2 data was assessed by simulating another 1.5-year gap in the exposure record when exposures were known, of the same duration as the existing gap. The effects of the imputed exposures were comparable to the effects of the known exposures (Sharpe,1999).

In most analyses testing for trends was carried out by representing the categories of exposure with ordinal variables, considered as continuous, and examining the significance of the coefficients with a z-test. *P*-values for trend for the analyses of the

*© 2000 Cancer Research Campaign*

stage 2 data were reported only in the final analyses, because matrix calculations were required (Cain and Breslow, 1988). We used $P < 0.05$ (two-sided) as the criterion of statistical significance.

### Confounder selection strategy

Confounders were selected using a strategy with both forward and backward components, decided upon *a priori* to avoid overadjustment (Day et al, 1980). Any variable representing a determinant of breast cancer whose inclusion in or deletion from a logistic model resulted in a 10% change in a RR associated with NSAID exposure was selected as a confounder (Mickey and Greenland, 1989). All RRs were adjusted for age.

### RESULTS

We accrued 5882 breast cancer cases and 23 517 controls. The cases' mean age at diagnosis was 64.1 years (13.3 s.d.).

Table 1 shows an analysis in which NSAID exposure was expressed as $\Sigma p_i$ for each period, controlling for exposure during the others. It included all subjects accrued from 1981 to 1995. Splitting the data into two groups by age at diagnosis (≤ 50 vs > 50 years) provided no evidence for effect modification by age at diagnosis (*P*-value for interaction = 0.82).

A protopathic bias was considered as an explanation for the trend towards increasing RRs associated with increasing NSAID exposure 7–12 months before the index date: pain from undiagnosed metastatic cancer might have resulted in NSAID exposure. The analysis of Table 1 was repeated, restricting it to cases without distant metastases at diagnosis, with almost identical results, suggesting that a protopathic bias was not involved.

Women with pain from any cause may have been examined, have had breast masses detected, and have received prescriptions for NSAIDs, producing a positive association between the dispensing of NSAIDs and the subsequent diagnosis of breast cancer. The analysis of Table 1 was repeated, restricting it to cases with tumours > 2 cm diameter at diagnosis, removing those with tumours more likely to have been detected by physicians' examinations and screening mammography. The RRs were very similar to those of Table 1, except for those for the two highest exposure levels 7–12 months earlier: they were reduced from 1.23 to 1.12 (95% CI 0.93–1.36) for $0.1<\Sigma p_i\leq 0.3$, and from 1.20 to 1.14 (95% CI 0.90–1.44) for $\Sigma p_i>0.3$, consistent with a detection bias.

To determine whether the trend towards decreasing RRs associated with increasing NSAID exposure 2–5 years earlier could be attributed to the effects of determinants of breast cancer associated with NSAID use, information on potential confounders was obtained by interviewing samples of subjects accrued from 30 June 1991 to 30 June 1995, selected according to both disease and exposure status.

Analyses like that of Table 1 were carried out using the data from the recently accrued stage 1 and stage 2 subjects (Sharpe, 1999). Of the stage 2 subjects 90.9% were post-menopausal on the index date, 6.0% were premenopausal, and 3.1% were of unknown menopausal status. The results of both analyses were adjusted only for age, exposure during the other time periods, and the sampling fractions. The results pertaining to stage 1 were similar to those of the overall analysis (Table 1). The results for stage 2 were also similar. However, the RRs associated with increasing exposure 1–6 months earlier ranged from 0.71–0.83, whereas the corresponding RRs for stage 1 ranged from 1.03–1.13. These differences could have been due to sampling error, to selection bias related to non-response, or both.

Consideration of selection bias was simplified because we knew NSAID exposure status, disease status and age for all subjects. We could examine the relationship between exposure and disease among all subjects and among subsets: those selected for interviews, those interviewed, and those who were not interviewed. We concluded that selection bias related to non-response was greatest for exposure during the 6-month period preceding the index date, and minimal in relation to earlier exposures (Sharpe, 1999).

To study confounding by drugs associated with breast cancer development we used data from the SPDP: there was no confounding by oral contraceptives, corticosteroids, or oestrogens. Nor was there confounding by the use of ibuprofen and aspirin bought over the counter, tobacco, alcohol, a positive family history of breast cancer among first-degree female relatives, the number of breast biopsies that resulted in benign diagnoses, educational attainment, age at menarche, age at first birth, height, body mass index (BMI) before menopause, or age at menopause.

However, the total duration of lactation and BMI after menopause were confounders. Table 2 juxtaposes the results of the stage 2 analysis without and with adjustment for these variables. Despite the wide 95% CIs and the large *P*-values for trend, Table 2 indicates the direction and magnitude of the confounding. The adjustment decreased the RRs associated with NSAID exposure 2–5 years earlier, whereas the RRs associated with the highest levels of exposure 6–10 and 11–15 years earlier became closer to the null.

In the remaining analyses, we studied the relationships between NSAID exposure and attributes of the cases' tumours at diagnosis.

We calculated ORs for tumour diameter > 5 cm diameter or tumour with direct extension to the chest wall or skin, including inflammatory carcinoma; these were designated as 'large' tumours. We adjusted simultaneously for exposure during all the time periods, age, and year of diagnosis. Table 3 (left panel) shows trends towards a decreasing OR for a large tumour associated with increasing NSAID exposure 2–5 and 6–10 years before diagnosis (*P*-trend = 0.056 and 0.02 respectively).

A similar analysis to determine the OR for breast cancer ≤ 2 cm diameter according to NSAID exposure found no trends.

Regional lymph node involvement by tumour at diagnosis was unassociated with NSAID exposure.

Table 3 (right panel) shows there was a trend towards an increasing OR for distant metastasis at diagnosis associated with increasing NSAID exposure 1–6 months before diagnosis (*P*-trend = 0.0001), whereas NSAID exposure 2–5 and 6–10 years before diagnosis was associated with trends towards decreasing ORs for distant metastasis at diagnosis (*P*-trend = 0.0003 and 0.03 respectively).

The increasing trend associated with NSAID exposure 1–6 months before diagnosis may have been to a protopathic bias, a detection bias, or both. The analysis was repeated after excluding the cases with primary tumours ≤ 2 cm diameter or unknown: the tumours most likely detected by systematic clinical examinations or by mammographic screening, which might be carried out more frequently among NSAID users. The results were very similar, suggesting that the trend was not due to a detection bias, leaving a protopathic bias as a possible explanation.

© 2000 Cancer Research Campaign

**Table 2** RRs of breast cancer according to NSAID exposure by time period before diagnosis for the stage 2 cases and controls (telephone interview respondents, accrued from 30 June 1991 through 30 June 1995). The RRs and the 95% CIs have been corrected for the sampling on both disease and exposure status. Exposure levels were imputed for those cases and controls whose exposure histories during the periods 2–5 and 6–10 years before the index date were interrupted by the 1.5-year gap in the records of the Prescription Drug Program database. As a result, the true 95% CIs are wider than those shown. The 'other' category of exposure, reserved for subjects with incomplete or missing exposure data, included some subjects during the period 11–15 years before the index date because of the dates upon which they entered the source population

| | NSAIDs | Stage 2 | | Crude | | Adjusted | |
|---|---|---|---|---|---|---|---|
| Period before diagnosis | (Average daily dose) | Cases *n* = 308 | Controls *n* = 309 | RR[a] | 95% CI | RR[b] | 95% CI |
| 1–6 months | 0 | 226 | 231 | 1.00 | Referent | 1.00 | Referent |
| | $0<\Sigma p_i\leq 0.1$ | 8 | 11 | 0.75 | 0.45–1.25 | 0.75 | 0.42–1.31 |
| | $0.1<\Sigma p_i\leq 0.3$ | 28 | 26 | 0.74 | 0.44–1.26 | 0.74 | 0.43–1.28 |
| | $\Sigma p_i>0.3$ | 46 | 41 | 0.84 | 0.44–1.62 | 0.81 | 0.42–1.58 |
| | *P* (trend) | | | | | | 0.54 |
| | Other | 0 | 0 | – | – | – | – |
| 7–12 months | 0 | 213 | 225 | 1.00 | Referent | 1.00 | Referent |
| | $0<\Sigma p_i\leq 0.1$ | 13 | 9 | 1.09 | 0.63–1.90 | 0.95 | 0.52–1.73 |
| | $0.1<\Sigma p_i\leq 0.3$ | 30 | 31 | 1.12 | 0.64–1.96 | 1.15 | 0.64–2.07 |
| | $\Sigma p_i>0.3$ | 52 | 44 | 1.26 | 0.61–2.60 | 1.26 | 0.60–2.63 |
| | *P* (trend) | | | | | | 0.52 |
| | Other | 0 | 0 | – | – | – | – |
| 2–5 years | 0 | 160 | 178 | 1.00 | Referent | 1.00 | Referent |
| | $0<\Sigma p_i\leq 0.1$ | 47 | 42 | 0.79 | 0.47–1.32 | 0.75 | 0.44–1.28 |
| | $0.1<\Sigma p_i\leq 0.3$ | 45 | 37 | 0.85 | 0.46–1.57 | 0.80 | 0.42–1.52 |
| | $\Sigma p_i>0.3$ | 56 | 52 | 0.87 | 0.41–1.83 | 0.82 | 0.38–1.77 |
| | *P* (trend) | | | | | | 0.89 |
| | Other | 0 | 0 | – | – | – | – |
| 6–10 years | 0 | 149 | 167 | 1.00 | Referent | 1.00 | Referent |
| | $0<\Sigma p_i\leq 0.1$ | 49 | 58 | 0.95 | 0.59–1.54 | 0.88 | 0.53–1.46 |
| | $0.1<\Sigma p_i>0.3$ | 56 | 41 | 1.08 | 0.62–1.87 | 1.00 | 0.56–1.78 |
| | $\Sigma p_i\leq 0.3$ | 54 | 43 | 1.27 | 0.65–2.49 | 1.06 | 0.52–2.14 |
| | *P* (trend) | | | | | | 0.56 |
| | Other | 0 | 0 | – | – | – | – |
| 11–15 years | 0 | 155 | 172 | 1.00 | Referent | 1.00 | Referent |
| | $0<\Sigma p_i\leq 0.1$ | 79 | 67 | 0.77 | 0.50–1.21 | 0.80 | 0.50–1.27 |
| | $0.1<\Sigma p_i>0.3$ | 42 | 40 | 0.87 | 0.53–1.43 | 0.92 | 0.55–1.53 |
| | $\Sigma p_i\leq 0.3$ | 18 | 20 | 0.58 | 0.30–1.11 | 0.72 | 0.37–1.42 |
| | *P* (trend) | | | | | | 0.58 |
| | Other | 14 | 10 | 1.09 | 0.75–1.59 | 1.03 | 0.68–1.54 |

[a]RRs adjusted for the sampling fractions, age and exposure during the other periods. [b]RRs adjusted for the sampling fractions, age, exposure during the other periods, total duration of lactation and BMI after menopause.

The apparent protective effect against distant metastasis associated with increasing NSAID exposure 2–5 and 6–10 years before diagnosis might be explained if NSAID users tended to be examined more regularly or receive mammography more frequently and have their primary tumours detected at a smaller size. However, as noted above, there was no association between NSAID exposure and having a primary tumour ≤ 2 cm diameter at diagnosis. In addition, deleting the subjects with tumours ≤ 2 cm diameter from the analysis did not remove the protective effect associated with increasing NSAID exposure 2–5 years before diagnosis (*P*-trend = 0.02). Finally, we repeated the analysis, controlling also for tumour size (≤ 2 cm diameter, 2–5 cm, > 5 cm or with extension). The protective effect associated with NSAID exposure 2–5 years before diagnosis remained, but the protective effect associated with exposure 6–10 years before diagnosis was no longer evident.

Screening mammography was introduced to Saskatchewan in 1990. Table 4 shows that there were trends towards decreasing relative odds of being diagnosed with a large tumour or with distant metastasis over time, after adjustment for age at diagnosis; *P*-trend = 0.004 and 0.052 respectively. These trends could have been due to earlier detection of breast cancer. However, after additional adjustment for NSAID use, the trends towards decreasing relative odds of being diagnosed with a large tumour or with distant metastasis over time were no longer evident; *P*-trend = 0.55 and 0.47 respectively. The ORs adjusted for NSAID use were derived from the analyses shown in Table 3, which show that NSAID exposure accounted for the relative odds of being diagnosed with a large tumour or with distant metastasis better than year of diagnosis.

### DISCUSSION

### Summary of findings

Increasing NSAID exposure was associated with an increasing RR for breast cancer 7–12 months later, which was attenuated by deleting the cases with the smallest tumours, consistent with a detection bias, rather than a true increase in risk. It was also associated with a decreasing RR for breast cancer 2–5 years later (Table 1), which could not be attributed to confounding by other determinants of breast cancer (Table 2).

 *© 2000 Cancer Research Campaign*

**Table 3** ORs for tumour size > 5 cm diameter or tumour with direct extension to the chest wall or skin ('large'), including inflammatory carcinoma, at diagnosis (left panel), and ORs for distant metastasis at diagnosis of breast cancer ('Mets (+)') (right panel), according to NSAID exposure by time period before diagnosis. The 'other' category was for subjects with incomplete or missing exposure information

| | **NSAIDs** | **Tumour size**[a] | | | | **Distant metastasis**[b] | | | |
|---|---|---|---|---|---|---|---|---|---|
| **Period before diagnosis** | **(Average daily dose)** | **Large *n* = 639** | **Small *n* = 4968** | **OR**[c] | **95% CI** | **Mets (+) *n* = 364** | **Mets (–) *n* = 5370** | **OR**[c] | **95% CI** |
| 1–6 months | 0 | 446 | 3273 | 1.00 | Referent | 204 | 3599 | 1.00 | Referent |
| | $0<\Sigma p_i\le 0.1$ | 30 | 271 | 0.89 | 0.60–1.34 | 23 | 289 | 1.61 | 1.01–2.57 |
| | $0.1<\Sigma p_i\le 0.3$ | 34 | 376 | 0.79 | 0.53–1.17 | 41 | 379 | 2.50 | 1.69–3.71 |
| | $\Sigma p_i>0.3$ | 44 | 381 | 0.99 | 0.62–1.57 | 39 | 395 | 2.79 | 1.68–4.64 |
| | *P* (trend) | | | | 0.64 | | | | 0.0001 |
| | Other | 85 | 667 | 0.91 | 0.63–1.32 | 57 | 708 | 1.12 | 0.71–1.76 |
| 7–12 months | 0 | 449 | 3235 | 1.00 | Referent | 225 | 3550 | 1.00 | Referent |
| | $0<\Sigma p_i\le 0.1$ | 31 | 263 | 0.95 | 0.63–1.41 | 22 | 277 | 1.15 | 0.71–1.86 |
| | $0.1<\Sigma p_i\le 0.3$ | 30 | 381 | 0.64 | 0.42–0.98 | 24 | 400 | 0.67 | 0.41–1.10 |
| | $\Sigma p_i>0.3$ | 44 | 391 | 0.84 | 0.51–1.37 | 30 | 411 | 0.62 | 0.35–1.12 |
| | *P* (trend) | | | | 0.32 | | | | 0.10 |
| | Other | 85 | 698 | 0.95 | 0.65–1.39 | 63 | 732 | 1.17 | 0.73–1.86 |
| 2–5 years | 0 | 212 | 1217 | 1.00 | Referent | 120 | 1364 | 1.00 | Referent |
| | $0<\Sigma p_i\le 0.1$ | 128 | 1075 | 0.74 | 0.58–0.94 | 60 | 1174 | 0.52 | 0.37–0.73 |
| | $0.1<\Sigma p_i\le 0.3$ | 30 | 269 | 0.75 | 0.48–1.15 | 18 | 287 | 0.53 | 0.30–0.92 |
| | $\Sigma p_i>0.3$ | 21 | 165 | 0.83 | 0.47–1.46 | 12 | 181 | 0.49 | 0.24–0.99 |
| | *P* (trend) | | | | 0.06 | | | | 0.0003 |
| | Other | 248 | 2242 | 0.69 | 0.54–0.90 | 154 | 2364 | 0.66 | 0.48–0.92 |
| 6–10 years | 0 | 147 | 898 | 1.00 | Referent | 86 | 991 | 1.00 | Referent |
| | $0<\Sigma p_i\le 0.1$ | 101 | 1052 | 0.62 | 0.47–0.82 | 69 | 1109 | 0.74 | 0.53–1.04 |
| | $0.1<\Sigma p_i\le 0.3$ | 20 | 208 | 0.61 | 0.37–1.01 | 11 | 220 | 0.47 | 0.24–0.92 |
| | $\Sigma p_i>0.3$ | 16 | 118 | 0.84 | 0.47–1.50 | 10 | 127 | 0.74 | 0.36–1.51 |
| | *P* (trend) | | | | 0.02 | | | | 0.03 |
| | Other | 355 | 2692 | 0.73 | 0.52–1.02 | 188 | 2923 | 0.88 | 0.57–1.34 |
| 11–15 years | 0 | 98 | 807 | 1.00 | Referent | 57 | 859 | 1.00 | Referent |
| | $0<\Sigma p_i\le 0.1$ | 83 | 795 | 0.92 | 0.68–1.26 | 34 | 846 | 0.62 | 0.40–0.96 |
| | $0.1<\Sigma p_i\le 0.3$ | 16 | 132 | 1.05 | 0.59–1.88 | 9 | 139 | 0.83 | 0.40–1.76 |
| | $\Sigma p_i>0.3$ | 5 | 56 | 0.67 | 0.25–1.75 | 5 | 58 | 1.03 | 0.38–2.74 |
| | *P* (trend) | | | | 0.63 | | | | 0.36 |
| | Other | 437 | 3178 | 1.02 | 0.54–1.94 | 259 | 3468 | 1.19 | 0.55–2.60 |

[a] 5,607 cases were included in this analysis; 275 cases were excluded because of missing tumour size information. [b] 5,734 cases were included in this analysis; 148 cases were excluded because of missing information regarding distant metastases. [c] ORs were calculated with unconditional logistic regression with adjustment for exposure during the other time periods, age at diagnosis, and year of diagnosis.

Increasing NSAID exposure was associated with a trend towards increasing ORs 1–6 months later for distant metastasis at diagnosis, probably due to a protopathic bias. However, it was also associated with trends towards decreasing ORs 2–5 and 6–10 years later for a 'large' breast cancer at diagnosis and for distant metastasis. NSAID exposure was unassociated with regional lymph node involvement.

### Representation of exposure

We represented exposure to NSAIDs as a class on the assumption that all NSAIDs have similar effects on the risk of breast cancer, since they all reduce prostaglandin synthesis by inhibiting cyclooxygenase (COX). However, COX exists as two isoenzymes: COX-1 is present in all cells, whereas COX-2 is induced by stimuli that provoke inflammation and by tumour promoters (Taketo, 1998). Although currently used NSAIDs inhibit both isoenzymes to varying extents, it is still unclear which is involved in tumourigenesis – COX-1, COX-2, or both. If one isoenzyme is more involved in tumourigenesis than the other, by analysing the effects of NSAIDs as a class any protective effects observed will be less extreme than the true effect of the most potent agent. Although COX-2 is induced in some breast cancers (Hwang et al, 1998), NSAIDs may have protective effects unrelated to COX inhibition (Thompson et al, 1995).

Since our measures of exposure were based on outpatient prescriptions, subjects' actual consumption of NSAIDs may have differed from our estimates. It is unlikely that all the drugs dispensed were ingested: we may have overestimated exposure. Alternatively, we may have underestimated exposure, since we had no information about NSAIDs dispensed in hospitals or as samples in physicians' offices. These amounts were probably small relative to the amounts we used in calculating exposure. The amounts of aspirin and ibuprofen bought over the counter were relatively small (Sharpe, 1999), and did not confound the effects of NSAIDs dispensed under the SPDP. However, subjects in the reference categories may have had low levels of exposure to either aspirin or ibuprofen or both.

*© 2000 Cancer Research Campaign*

**Table 4** ORs for tumour size and for distant metastasis according to year of diagnosis, with adjustment for age at diagnosis, or for age at diagnosis and NSAID exposure as represented in Table 3. 'Large' indicates tumour size > 5 cm diameter or tumour with direct extension to the chest wall or skin, including inflammatory carcinoma. The ORs and their 95% CIs in the two columns on the right side of the table were derived from the analyses shown in Table 3

| Year of diagnosis | Large *n* = 639 | Small *n* = 4968 | Adjusted for age | | Adjusted for age and NSAID Use | |
|---|---|---|---|---|---|---|
| | | | OR[a] | 95% CI | ORa | 95% CI |
| 1981–1985 | 203 | 1363 | 1.00 | Referent | 1.00 | Referent |
| 1986–1990 | 222 | 1701 | 0.86 | 0.70–1.06 | 0.92 | 0.64–1.33 |
| 1991–1995 | 214 | 1904 | 0.74 | 0.60–0.90 | 0.86 | 0.46–1.63 |
| *P* (trend) | | | | 0.004 | | 0.55 |
| | **Mets (+) n = 364** | **Mets (–) n = 5370** | | | | |
| 1981–1985 | 106 | 1517 | 1.00 | Referent | 1.00 | Referent |
| 1986–1990 | 145 | 1830 | 1.09 | 0.84–1.42 | 1.17 | 0.73–1.88 |
| 1991–1995 | 113 | 2023 | 0.77 | 0.59–1.02 | 1.22 | 0.56–2.66 |
| *P* (trend) | | | | 0.052 | | 0.47 |

[a] Calculated with unconditional logistic regression.

If we overestimated NSAID exposure among those subjects classified as highly exposed and underestimated NSAID exposure among those subjects classified as unexposed, then the slopes of the dose–risk relationships that we observed would be less extreme than the true slope (MacMahon and Trichopoulos, 1996). However, the most highly exposed subjects may have ingested almost all of the NSAIDs dispensed, if they suffered chronic pain. If we accurately estimated overall NSAID exposure among the highly exposed and underestimated it among the nonexposed, then the true dose–risk curves would be shifted to the right relative to what we observed with increased slopes.

### Other potential confounders

Could there have been confounding by other known determinants of breast cancer that we did not measure?

We did not collect information on subjects' indications for NSAID use, because no associations have been reported with breast cancer. However, heavy users of NSAIDs with chronic arthritis could have been less physically active than those less highly exposed. Although physical activity has been associated with protective effects (Thune et al, 1997), we did not measure it because it is not yet clear that the protective effects are due to physical activity per se rather than uncontrolled confounding or recall bias (Gammon et al, 1998). If physical activity is protective and if the heaviest users of NSAIDs were physically inactive, then they would have been at higher risk of developing breast cancer. The true protective effects of NSAID exposure on risk may be more extreme than those we observed.

Our analyses of the effects of NSAID exposure on stage could be confounded by other determinants of tumour stage associated with NSAID exposure. Since lower socioeconomic status and obesity tend to be associated with both NSAID exposure (Sharpe, 1999) and with higher stage at diagnosis (Richardson et al, 1992; Hunter et al, 1993), these variables may have been confounders. If NSAID users were at increased risk of presenting with higher stage disease, then the true protective effects of NSAIDs against large tumour size and distant metastasis may be more extreme than we observed.

### Interpretation of results

It is unlikely that the dose-dependent reduction in the RR for breast cancer associated with NSAID exposure 2–5 years earlier was due to chance (Table 1). Selection or recall bias were unlikely due to the study design. If the pattern of confounding among the subjects in the overall analysis (Table 1) was similar to that observed among the stage 2 subjects (Table 2), then this protective effect of NSAID exposure cannot be attributed to confounding. A biologic explanation is called for.

Walter and Day (1983) estimated the duration of the detectable preclinical phase of breast cancers with data from a mammographic screening trial. Their results suggest that 5–56% of our cases had already developed mammographically detectable breast cancers during the period 2–5 years before the index date, if one assumes that the source population was not systematically screened. However, the introduction of mammographic screening to Saskatchewan began in 1990, so this estimate may be high. Nevertheless, it is unlikely that NSAIDs prevent breast cancer from developing de novo, since time must elapse between tumour initiation and mammographic detectability. NSAIDs may slow the growth of tumours, reducing the probability of detection on the index date; i.e. postponing it.

Experimental results support this conclusion. Lala et al (1997) found that the oral administration of indomethacin to a strain of mice, 90% of which develop spontaneous mammary carcinomas, delayed the appearance of the first tumours by 11–12 weeks. The tumours of the treated mice showed increased cell death and reduced vascularity. Robertson et al (1998) administered ibuprofen to rats that developed mammary carcinomas after receiving a carcinogen 100 days earlier. After 35 days of ibuprofen, the tumour volumes of the treated animals had decreased, whereas those of the controls had increased, showing that NSAIDs can reverse the growth of established mammary carcinomas.

 *© 2000 Cancer Research Campaign*

The magnitude of the protective effect on risk that we observed at the highest level of exposure (Table 1, RR = 0.76, 95% CI 0.63–0.92) was comparable to that observed by others (Gridley et al, 1993; Schreinemachers and Everson, 1994; Friedman and Ury, 1980; Harris et al, 1995, 1996). However, some did not find similar effects. Some studies used only limited exposure data (Isömaki et al, 1978; Thun et al, 1993). All failed to deal with the timing of exposure, which might explain why Egan et al (1996) found that aspirin had no effect.

The fact that the protective effects of NSAIDs on incidence, tumour size and risk of distant metastasis were all associated with exposure 2–5 years before diagnosis suggests that NSAIDs may have interfered with aspects of tumour development which occurred during that period.

NSAIDs could slow or reverse the growth of both primary tumours and distant metastases by promoting apoptosis (Han et al, 1998; Lala et al, 1997) and inhibiting angiogenesis (Ziche et al, 1982; Hudson et al, 1995; Lala et al, 1997). For example, indomethacin treatment reduced the incidence of macroscopic metastatic lung nodules that developed in athymic nude mice after subcutaneous transplantation of a human breast cancer cell line, but had no effect on the incidence of micrometastases. Indomethacin may have interfered with the phase of tumour growth dependent on angiogenesis and prevented micrometastases from becoming macroscopic (Connolly et al, 1996).

The onset of tumour neovascularization coincides with the spread of tumour cells into the circulation and the development of distant metastases (Folkman, 1995). These processes require tumour cells to invade the vascular endothelium on entering and leaving the circulation. NSAIDs may interfere with this process in vivo, because in vitro indomethacin suppresses the invasion of human breast cancer cells through a reconstituted basement membrane (Connolly and Rose, 1993).

NSAIDs may have had no effect on the spread of breast cancer to the regional lymph nodes because angiogenesis and vascular invasion were not involved in its occurrence.

### CONCLUSION

The magnitude of the protective effects associated with NSAID exposure that occur 2–5 years later, the existence of dose–risk relationships, and the coherence of these results with the experimental evidence strongly suggest that NSAIDs may slow the growth of established but undiagnosed breast cancers and reduce the risk of distant metastasis at diagnosis.

Although trials of chemoprevention by NSAIDs are being advocated against breast cancer (Vainio and Morgan, 1998), our interpretation suggests that trials involving women screened at entry might not demonstrate protective effects. NSAIDs may have more potential as therapeutic agents than as preventive agents: they may be useful in delaying or preventing the appearance of distant metastases after diagnosis.

### ACKNOWLEDGEMENTS

This project was funded by a grant (no. 007312) from the Canadian Breast Cancer Research Initiative. Dr Sharpe is supported by a postdoctoral fellowship from the Medical Research Council of Canada and Dr Collet by a grant from the Fonds de la Recherche en Santé du Québec. The study was based in part on data provided by the Saskatchewan Department of Health. The interpretation and conclusions contained in this report do not necessarily represent those of the Government of Saskatchewan or the Saskatchewan Department of Health. We are grateful to the following employees of Saskatchewan Health: Patty Beck, Leah Lueck, Konnie Malowany, Julie Anton, Collette Forbes, Michelle Manson, Brenda Saunders and Glinda Smith.

The study was also based on data provided by the Saskatchewan Cancer Agency, which bears no responsibility for the analysis of the data or the interpretation of the results. We are grateful to the following employees of the Saskatchewan Cancer Agency: Diane Robson, Judy Kosloski, Norman Brown, Darryl Salisbury, Sharon Luterback and Carla Leibel.

### REFERENCES

Breslow NE and Day NE (1980) *Statistical Methods in Cancer Research. Vol. I – The Analysis of Case–control Studies*. International Agency for Research on Cancer: Lyon

Cain KC and Breslow NE (1988) Logistic regression analysis and efficient design for two-stage studies. *Am J Epidemiol* **128:** 1198–1206

Collet J-P, Sharpe C, Belzile E, Boivin J-F, Hanley J and Abenhaim L (1999) Colorectal cancer prevention by non-steroidal anti-inflammatory drugs: effects of dosage and timing. *Br J Cancer* **81:** 62–68

Connolly JM and Rose DP (1993) Effects of fatty acids on invasion through reconstituted basement membrane (‘Matrigel’) by a human breast cancer cell line. *Cancer Lett* **75:** 137–142

Connolly JM, Liu XH and Rose DP (1996) Dietary linoleic acid-stimulated human breast cancer cell growth and metastasis in nude mice and their suppression by indomethacin, a cyclooxygenase inhibitor. *Nutr Cancer* **25:** 231–240

Day NE, Byar DP and Green SB (1980) Overadjustment in case–control studies. *Am J Epidemiol* **112:** 696–706

Egan KM, Stampfer MJ, Giovannucci E, Rosner BA and Colditz GA (1996) Prospective study of regular aspirin use and the risk of breast cancer. *J Natl Cancer Inst* **88:** 988–993

Folkman J (1995) The influence of angiogenesis research on management of patients with breast cancer. *Breast Cancer Res Treat* **36:** 109–118

Friedman GD and Ury HK (1980) Initial screening for carcinogenicity of commonly used drugs. *J Natl Cancer Inst* **65:** 723–733

Gammon MD, John EM and Britton JA (1998) Recreational and occupational physical activities and risk of breast cancer. *J Natl Cancer Inst* **90:** 100–117

Gridley G, McLaughlin JK, Ekbom A, Klareskog L, Adami HO, Hacker DG, Hoover R and Fraumeni JF (1993) Incidence of cancer among patients with rheumatoid arthritis. *J Natl Cancer Inst* **85:** 307–311

Han EK, Arber N, Yamamoto H, Lim JT, Delohery T, Pamukcu R, Piazza GA and Xing WQ (1998) Effects of sulindac and its metabolites on growth and apoptosis in human mammary epithelial and breast carcinoma cell lines. *Breast Cancer Res Treat* **48:** 195–203

Harris RE, Namboodiri KK, Stellman SD and Wynder EL (1995) Breast cancer and NSAID use: heterogeneity of effect in a case–control study. *Prev Med* **24:** 119–120

Harris RE, Namboodiri KK and Farrar WB (1996) Non-steroidal anti-inflammatory drugs and breast cancer. *Epidemiology* **7:** 203–205

Hubbard NE, Chapkin RS and Erickson KL (1988) Inhibition of growth and linoleate-enhanced metastasis of a transplantable mouse mammary tumor by indomethacin. *Cancer Lett* **43:** 111–120

Hudson N, Balsitis M, Everitt S and Hawkey CJ (1995) Angiogenesis in gastric ulcers: impaired in patients taking non-steroidal anti-inflammatory drugs. *Gut* **37:** 191–194

Hunter CP, Redmond CK, Chen VW, Austin DF, Greenberg RS, Correa P, Muss HB and Forman MR, Wesley MN, Blacklow RS, Kurman RJ, Dignam JJ, Edwards BK and Shapiro S, and other members of the Black/White Cancer Survival Study Group (1993) Breast cancer: factors associated with stage at diagnosis in black and white women. *J Natl Cancer Inst* **85:** 1129–1137

Hwang D, Scollard D, Byrne J and Levine E (1998) Expression of cyclooxygenase-1 and cyclooxygenase-2 in human breast cancer. *J Natl Cancer Inst* **90:** 455–460

Isomäki HA, Hakulinen T and Joutsenlahti U (1978) Excess risk of lymphomas, leukemia and myeloma in patients with rheumatoid arthritis. *J Chron Dis* **31:** 691–696

*© 2000 Cancer Research Campaign*

Khoo NKS, Chan FPH, Saarloos MN and Lala PK (1992) Immunotherapy of mammary adenocarcinoma metastases in C3H/HeN mice with chronic administration of cyclooxygenase inhibitors alone or in combination with IL-2. *Clin Exp Metastasis* **10:** 239–252
Krogh CME (ed) (1995) *Compendium of Pharmaceuticals and Specialities*, 30th edn. Canadian Pharmaceutical Association: Ottawa
Lala PK, Al-Mutter N and Orucevic A (1997) Effects of chronic indomethacin therapy on the development and progression of spontaneous mammary tumors in C3H/HEJ mice. *Int J Cancer* **73:** 371–380
MacMahon B and Trichopoulos D (1996) *Epidemiology. Principles and Methods*, 2nd edn. Little, Brown: Boston
Mickey RM and Greenland S (1989) The impact of confounder selection criteria on effect estimation. *Am J Epidemiol* **129:** 125–137
Miettinen OS (1985) *Theoretical Epidemiology. Principles of Occurrence Research in Medicine*. Delmar: Albany
Parkin DM, Whelan SL, Ferlay J, Raymond L and Young J (eds) (1997) *Cancer Incidence in Five Continents*, Vol. VII. International Agency for Research on Cancer: Lyon
Rawson NSB, D'Arcy C, Blackburn JL, Bowen RC, Wallace SM, Downey W and Senft D (1992) Epidemiologic research using linked computerized health care datafiles in Saskatchewan, Canada. Technical report series. In *Report #2. Psychiatric Pharmacoepidemiology Research Consortium*: Saskatoon
Richardson JL, Langholz B, Bernstein L, Burciaga C, Danley K and Ross RK (1992) Stage and delay in breast cancer diagnosis by race, socioeconomic status, age and year. *Br J Cancer* **65:** 922–926
Risch HA and Howe GR (1994) Menopausal hormone usage and breast cancer in Saskatchewan: a record linkage study. *Am J Epidemiol* **139:** 670–683
Robertson FM, Parrett ML, Joarder FS, Ross M, Abou-Issa HM, Alshafie G and Harris RE (1998) Ibuprofen-induced inhibition of cyclooxygenase isoform gene expression and regression of rat mammary carcinomas. *Cancer Lett* **122:** 165–175
Schreinemachers DM and Everson RB (1994) Aspirin use and lung, colon and breast cancer incidence in a prospective study. *Epidemiology* **5:** 138–146
Sharpe CR (1999) *Population-based Case–control Study of the Effects of Non-steroidal Anti-inflammatory Drugs on the Risk of Breast Cancer*. PhD thesis. McGill University: Montréal
Spiessl B, Beahrs OH, Hermanek P, Hutter RVP, Scheibe O, Sobin LH and Wagner G, editors (1992) *TNM Atlas. Illustrated Guide to the TNM/pTNM Classification of Malignant Tumours*, 3rd edn, 2nd revision. Springer-Verlag: Berlin
Taketo MM (1998) Cyclooxygenase-2 inhibitors in tumorigenesis (Part I). *J Natl Cancer Inst* **90:** 1529–1536
Thompson HJ, Briggs S, Paranka NS, Piazza GA, Brendel K, Gross PH, Sperl GJ, and Pamukcu R (1995) Inhibition of mammary carcinogenesis in rats by sulfone metabolite of sulindac. *J Natl Cancer Inst* **87:** 1259–1260
Thun MJ, Namboodiri MM, Calle EE, Flanders WD and Heath CW (1993) Aspirin use and risk of fatal cancer. *Cancer Res* **53:** 1322–1327
Thune I, Brenn T, Lund E and Gaard M (1997) Physical activity and the risk of breast cancer. *N Engl J Med* **336:** 1269–1275
Vainio H and Morgan G (1998) Cyclooxygenase 2 and breast cancer prevention. *Br Med J* **317:** 828
Walter SD and Day NE (1983) Estimation of the duration of a preclinical disease state using screening data. *Am J Epidemiol* **118:** 865–886
Ziche M, Jones J and Gullino PM (1982) Role of prostaglandin $E_1$ and copper in angiogenesis. *J Natl Cancer Inst* **69:** 475–482

 *© 2000 Cancer Research Campaign*

## References

[PDF_00798] Cain K. C., Breslow N. E. (1988). Logistic regression analysis and efficient design for two-stage studies.. Am J Epidemiol.

[PDF_00800] Collet J. P., Sharpe C., Belzile E., Boivin J. F., Hanley J., Abenhaim L. (1999). Colorectal cancer prevention by non-steroidal anti-inflammatory drugs: effects of dosage and timing.. Br J Cancer.

[PDF_00806] Connolly J. M., Liu X. H., Rose D. P. (1996). Dietary linoleic acid-stimulated human breast cancer cell growth and metastasis in nude mice and their suppression by indomethacin, a cyclooxygenase inhibitor.. Nutr Cancer.

[PDF_00803] Connolly J. M., Rose D. P. (1993). Effects of fatty acids on invasion through reconstituted basement membrane ('Matrigel') by a human breast cancer cell line.. Cancer Lett.

[PDF_00809] Day N. E., Byar D. P., Green S. B. (1980). Overadjustment in case-control studies.. Am J Epidemiol.

[PDF_00811] Egan K. M., Stampfer M. J., Giovannucci E., Rosner B. A., Colditz G. A. (1996). Prospective study of regular aspirin use and the risk of breast cancer.. J Natl Cancer Inst.

[PDF_00814] Folkman J. (1995). The influence of angiogenesis research on management of patients with breast cancer.. Breast Cancer Res Treat.

[PDF_00816] Friedman G. D., Ury H. K. (1980). Initial screening for carcinogenicity of commonly used drugs.. J Natl Cancer Inst.

[PDF_00818] Gammon M. D., John E. M., Britton J. A. (1998). Recreational and occupational physical activities and risk of breast cancer.. J Natl Cancer Inst.

[PDF_00820] Gridley G., McLaughlin J. K., Ekbom A., Klareskog L., Adami H. O., Hacker D. G., Hoover R., Fraumeni J. F. (1993). Incidence of cancer among patients with rheumatoid arthritis.. J Natl Cancer Inst.

[PDF_00823] Han E. K., Arber N., Yamamoto H., Lim J. T., Delohery T., Pamukcu R., Piazza G. A., Xing W. Q., Weinstein I. B. (1998). Effects of sulindac and its metabolites on growth and apoptosis in human mammary epithelial and breast carcinoma cell lines.. Breast Cancer Res Treat.

[PDF_00830] Harris R. E., Namboodiri K. K., Farrar W. B. (1996). Nonsteroidal antiinflammatory drugs and breast cancer.. Epidemiology.

[PDF_00827] Harris R. E., Namboodiri K., Stellman S. D., Wynder E. L. (1995). Breast cancer and NSAID use: heterogeneity of effect in a case-control study.. Prev Med.

[PDF_00832] Hubbard N. E., Chapkin R. S., Erickson K. L. (1988). Inhibition of growth and linoleate-enhanced metastasis of a transplantable mouse mammary tumor by indomethacin.. Cancer Lett.

[PDF_00835] Hudson N., Balsitis M., Everitt S., Hawkey C. J. (1995). Angiogenesis in gastric ulcers: impaired in patients taking non-steroidal anti-inflammatory drugs.. Gut.

[PDF_00838] Hunter C. P., Redmond C. K., Chen V. W., Austin D. F., Greenberg R. S., Correa P., Muss H. B., Forman M. R., Wesley M. N., Blacklow R. S. (1993). Breast cancer: factors associated with stage at diagnosis in black and white women. Black/White Cancer Survival Study Group.. J Natl Cancer Inst.

[PDF_00843] Hwang D., Scollard D., Byrne J., Levine E. (1998). Expression of cyclooxygenase-1 and cyclooxygenase-2 in human breast cancer.. J Natl Cancer Inst.

[PDF_00845] Isomäki H. A., Hakulinen T., Joutsenlahti U. (1978). Excess risk of lymphomas, leukemia and myeloma in patients with rheumatoid arthritis.. J Chronic Dis.

[PDF_00850] Khoo N. K., Chan F. P., Saarloos M. N., Lala P. K. (1992). Immunotherapy of mammary adenocarcinoma metastases in C3H/HeN mice with chronic administration of cyclo-oxygenase inhibitors alone or in combination with IL-2.. Clin Exp Metastasis.

[PDF_00856] Lala P. K., Al-Mutter N., Orucevic A. (1997). Effects of chronic indomethacin therapy on the development and progression of spontaneous mammary tumors in C3H/HEJ mice.. Int J Cancer.

[PDF_00861] Mickey R. M., Greenland S. (1989). The impact of confounder selection criteria on effect estimation.. Am J Epidemiol.

[PDF_00872] Richardson J. L., Langholz B., Bernstein L., Burciaga C., Danley K., Ross R. K. (1992). Stage and delay in breast cancer diagnosis by race, socioeconomic status, age and year.. Br J Cancer.

[PDF_00875] Risch H. A., Howe G. R. (1994). Menopausal hormone usage and breast cancer in Saskatchewan: a record-linkage cohort study.. Am J Epidemiol.

[PDF_00877] Robertson F. M., Parrett M. L., Joarder F. S., Ross M., Abou-Issa H. M., Alshafie G., Harris R. E. (1998). Ibuprofen-induced inhibition of cyclooxygenase isoform gene expression and regression of rat mammary carcinomas.. Cancer Lett.

[PDF_00881] Schreinemachers D. M., Everson R. B. (1994). Aspirin use and lung, colon, and breast cancer incidence in a prospective study.. Epidemiology.

[PDF_00889] Taketo M. M. (1998). Cyclooxygenase-2 inhibitors in tumorigenesis (part I).. J Natl Cancer Inst.

[PDF_00891] Thompson H. J., Briggs S., Paranka N. S., Piazza G. A., Brendel K., Gross P. H., Sperl G. J., Pamukcu R., Ahnen D. J. (1995). Inhibition of mammary carcinogenesis in rats by sulfone metabolite of sulindac.. J Natl Cancer Inst.

[PDF_00894] Thun M. J., Namboodiri M. M., Calle E. E., Flanders W. D., Heath C. W. (1993). Aspirin use and risk of fatal cancer.. Cancer Res.

[PDF_00896] Thune I., Brenn T., Lund E., Gaard M. (1997). Physical activity and the risk of breast cancer.. N Engl J Med.

[PDF_00898] Vainio H., Morgan G. (1998). Cyclo-oxygenase 2 and breast cancer prevention. Non-steroidal anti-inflammatory agents are worth testing in breast cancer.. BMJ.

[PDF_00900] Walter S. D., Day N. E. (1983). Estimation of the duration of a pre-clinical disease state using screening data.. Am J Epidemiol.

[PDF_00902] Ziche M., Jones J., Gullino P. M. (1982). Role of prostaglandin E1 and copper in angiogenesis.. J Natl Cancer Inst.

